# Psychoactive substances and previous hospital admissions, triage and length of stay in rural injuries: a prospective observational study

**DOI:** 10.1186/s13049-023-01156-z

**Published:** 2023-11-27

**Authors:** Thomas Wilson, Torben Wisborg, Vigdis Vindenes, Ragnhild Elèn Gjulem Jamt, Stig Tore Bogstrand

**Affiliations:** 1 Faculty of Health Sciences, University of Tromsø, The Arctic University of Norway, PO Box 6050, 9037 Tromsø, Norway; 2 Department of Forensic Sciences, Section for Drug Abuse Research, Oslo University Hospital, Lovisenberggaten 6, 0456 Oslo, Norway; 3 Department of Anaesthesia and Intensive Care, Hammerfest Hospital, Finnmark Hospital Trust, 9601 Hammerfest, Norway; 4 Norwegian National Advisory Unit on Trauma, Division of Emergencies and Critical Care, Oslo University Hospital, PO box 4950, 0424 Nydalen, Oslo, Norway; 5 Centre of Laboratory Medicine, Østfold Hospital, PO Box 300, 1714 Grålum, Norway; 6 Institute of Health and Society, Faculty of Medicine, University of Oslo, PO Box 1078, 0316 Blindern, Oslo, Norway; 7 Department of Nursing and Health Promotion, Acute and Critical Illness, Faculty of Health Sciences, OsloMet - Oslo Metropolitan University, 0130 Oslo, Norway

**Keywords:** Psychoactive substances, Injury severity, MAIS, Repeat admittance, Duration, Rural, Resource, Intensive care

## Abstract

**Background:**

Patients admitted to hospital after an injury are often found to have used psychoactive substances prior to the injury. The aim of this study was to investigate the associations between psychoactive substances (alcohol, psychoactive medicinal drugs and illicit drugs) and previous hospital admissions, triage and length of stay in the arctic Norwegian county of Finnmark.

**Methods:**

Patients ≥ 18 years admitted due to injury to trauma hospitals in Finnmark from January 2015 to August 2016 were approached. Parameters regarding admittance and hospital stay were collected from 684 patients and blood was analysed for psychoactive substances. Using a prospective, observational design, time, triage, length of stay in hospital, use of intensive care unit (ICU), injury severity, Alcohol Use Disorder Identification Test—Consumption (AUDIT-C) and number of previous admittances were investigated by bivariable testing and logistical regression analysis.

**Results:**

Of 943 patients approached, 81% consented and 684 were included in the study. During the weekend, 51.5% tested positive for any substance versus 27.1% Monday–Friday. No associations were identified between testing positive and either triage or injury severity for any substance group although triage level was lower in patients with AUDIT-C ≥ 5. Short length of stay was associated with alcohol use prior to injury [odds ratio (OR) 0.48 for staying > 12 h, confidence interval (CI) 0.25–0.90]. The OR for staying > 24 h in the ICU when positive for an illicit substance was 6.33 (CI 1.79–22.32) while negatively associated with an AUDIT-C ≥ 5 (OR 0.30, CI 0.10–0.92). Patients testing positive for a substance had more often previously been admitted with the strongest association for illicit drugs (OR 6.43 (CI 1.47–28.08), compared to patients in whom no substances were detected.

**Conclusions:**

Triage level and injury severity were not associated with psychoactive substance use. Patients using alcohol are more often discharged early, but illicit substances were associated with longer ICU stays. All psychoactive substance groups were associated with having been previously admitted.

## Background

The use of alcohol, psychoactive prescription drugs and illicit narcotics is prevalent in injured adult emergency department (ED) patients, approaching 36% in our rural hospitals [1]. The consequences of alcohol use prior to injury on hospital resource consumption and outcomes vary, in some groups increasing injury severity and resource burden, e.g. in bicyclists [2], in burn patients prolonging hospital stays and worsening outcomes [3] and is associated with higher rates of acute respiratory distress syndrome (ARDS) [4]. A review of major trauma showed no overall effect on length of stay or need for intensive care unit (ICU) treatment with alcohol use, albeit with marginal effects on complications [5], and in a broader trauma population, no overall effect of a positive blood alcohol level on injury severity or length of stay was found albeit with both positive and negative correlations in sub-groups [6].

Both positive and negative effects of medicinal and illicit drugs on resource consumption after injury have been identified [7]. Sub-groups of illicit substance users have increased prevalence of pulmonary insufficiency and length of stay [8] while cannabis increases complications after burn injuries [9]. Z-hypnotics can increase length of stay in the elderly [10] and opioids in patients aged over 50 increased odds of having more ED visits and longer hospital stays [11]. The Alcohol Use Disorder Identification Test—Consumption (AUDIT-C) is a brief questionnaire used to identify risky drinking and is associated with testing positive for a psychoactive substance after injury [1]. An increased AUDIT-C is associated with increased mortality and occurrence of trauma [12] but little data exists on its correlation with resource consumption after injury.

In a Swedish ED, recurrent injuries were reported in 36% of trauma patients [13], and it is known that alcohol, psychoactive medicinal drugs and illicit substances increase the risk of recurrent trauma [14, 15]. However, data on whether other hospital admissions than trauma indicate risk of later trauma is lacking.

Trauma outcomes in rural areas are complicated by particular challenges and increased mortality, preventive measures are therefore important [16–19]. Many studies focus on varying groups, such as major trauma or sub-groups of psychoactive substances; and little is still known about rural use of healthcare prior to the injury. This is an analysis of a data set from the two rural emergency hospitals in the northernmost county in Norway gathered in 2015–2016.

Present research varies in whether psychoactive substance use impacts readily identifiable factors indicating resource burden in hospital. Our aim was to identify the association between psychoactive substances and hospital use in patients admitted due to injury in Finnmark County. Largely rural, it is the largest county in Norway covering an area larger than Denmark while inhabiting only 76.000 people. Patients admitted to hospital for injury are in rural Norway to a high extent examined and triaged to hospital by an on-call primary care physician. Primary analyses were presenting time of day, length of hospital stay, use of intensive care services and injury severity. Secondary analyses were number of previous admittances to hospital vs AUDIT-C. The aim of this study was to explore if injured psychoactive substance users consume more hospital services.

## Methods

### Participants and study design

The prospective observational study this paper is based on included 684 participants ≥ 18 years of age admitted due to injury to the two hospitals in Finnmark county from January 2015 to August 2016, based on their informed consent. Injuries unrelated to an accident were excluded, as were patients unable to consider informed consent due to pre-existing conditions or lasting incapacitation. Verbal and written information was provided before consent could be given, a blood sample was drawn and a questionnaire was filled out by the participant. Blood samples were analysed for psychoactive substances at the Department of Forensic Sciences, Oslo University Hospital, Norway by headspace gas chromatographic flame ionization detection (ethanol) and ultra-high performance liquid chromatography tandem mass spectrometry (drugs). The data collection and blood sample analysis is described in detail in a previous paper from the same study [1].

### Sample size

We did not perform a formal power analysis, but earlier studies indicate that the number of participants needed to provide acceptable statistical strength would be available over a 1.5 year period [20].

### Variables

Blood samples and self-reported alcohol use were collected. Main categories were ethanol with or without self-reported use, psychoactive medicinal drugs (opioids, sedatives and hypnotics) and illicit substances. Benzodiazepines such as flunitrazepam and phenazepam are kept in the group psychoactive medicinal substances due to Finnmark’s border with Russia where such benzodiazepines at least until recently have been more common in (legal) use. Hospital use measured by previous admittances and length of stay was utilized as indicators of specialist healthcare use. Admittance time of day could be different to urban areas due to often long transport distances, so as to identify any possible focus on timing of preventive efforts.

To account for prehospital delays caused by rurality, self-reported use of alcohol was for relevant analyses included in the definition of a positive result and has been shown to be representative [1]. Analysed substances are listed in Table 1. The questionnaire provided: triage level on admittance, the participants’ own indication of having used alcohol within the 6 h preceding the incident, and AUDIT-C. Length of hospital stay, ICU stay and previous admittance to the Finnmark hospital trust was subsequently acquired from the participants’ medical records by project staff. AUDIT-C was used to identify at-risk drinking, a cut-off score of ≥ 5 identified as an optimal cut-off level for identifying hazardous alcohol use among trauma patients [21]. Triage level was per the Medical Emergency Triage and Treatment System (METTS) [22], with red and orange triage giving the highest suspicion of serious injury. Red triage would require an emergency team activation or doctor immediately on arrival, and orange the presence of a doctor within 20 min. Symptoms and vital signs define colour, and remaining groups (yellow, green, blue) allow longer waiting times in the ED for a doctor’s evaluation [2 h and above]. Blood sample findings were corrected for medications administered after the incident. In this study, serious injury is defined as Maximum Abbreviated Injury Scale (MAIS) 3 and above, as defined by the European Commission and the High Level Group on Road Safety [23] and Injury Severity Score (ISS) ≥ 9 [24]. In coding of MAIS and ISS, The Abbreviated Injury Scale (AIS) ICD-ISS map v. 1.1, and ICD10 to ISS maps were used [25, 26].

**Table 1:** Test panel, identified substances

	Substance	Cut-off (µmol/l unless otherwise specified)
Alcohol	Ethanol	0.1 g/l
Psychoactive medicinal drugs	Alprazolam	0.01
	Diazepam	0.2
	Phenazepam	0.005
	Flunitrazepam	0.005
	Clonazepam	0.004
	Oxazepam	0.6
	Codeine	0.03
	Morphine	0.03
	7-aminonitrazepam	0.05
	Nitrazepam	0.05
	Zolpidem	0.07
	Zopiclone	0.02
Illicit substances	Amphetamine	0.2
	Benzoylecgonine	0.2
	Methamphetamine	0.2
	Tetrahydrocannabidiol	0.002

Table 1 shows the investigated substances in each group with cut-off values

The following substances was tested for but not identified: 5F-APINACA, 5F-PB-22, 6-monoacetylmorphine, 7-aminoflunitrazepam, 7-aminoclonazepam, alpha-PVP, buprenorphine, cocaine, etizolam, flubromazepam, LSD, MDMA (ecstasy), methadone, metiopropamine, meprobomate, n-desmethyldiazepam, phenobarbital

## Statistical analyses

For statistical analysis, IBM^®^ SPSS^®^ Statistics 26 was used. Bivariable cross tables with Pearson’s Chi^2^ analysis were used to identify associations. If necessary, Fischer’s exact test was employed. To further investigate risk factors we used multivariable logistic regression models, correcting for sex and age, and also injury severity where appropriate. Age groupings were 18–34, 35–64 and > 64 years of age, attempting similarity to previous studies. Level of significance was set at *p* < 0.05 and the Strengthening the Reporting of Observational studies in Epidemiology (STROBE) guideline was used to support quality of reporting [27]. Data monitoring was performed by the authors of this paper on registration, with double control of all data transferred from questionnaires into the database at regular intervals and with continuous follow-up of quality and ethical considerations. The data was stored at dedicated research data servers according to the guidelines of Oslo University Hospital.

### Study population

**Fig. 1:**
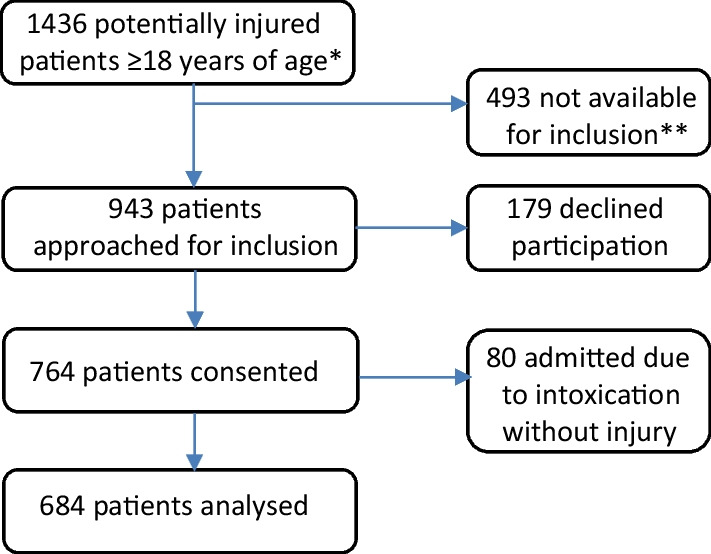
Flow chart showing patient inclusion into the study. Inclusion and exclusion of patients considered for study participation. *1436 patients were admitted to the Emergency Department due to a primary suspicion of injury. **493 patients were unavailable for approach due to reduced ability to consider consent, language barriers, death during admittance, failed blood sampling, direct transfer to intensive care or regional trauma centre or being unavailable for information or later follow-up

Included in the study were 684 consenting patients. A flow chart adapted from a previous article describing the prevalence of psychoactive substance use in injured patients and details about the data collection is shown in Fig. 1 [1].

## Results

Of 943 patients considered for inclusion, 179 (19%) declined participation. Of the remaining 764, 80 (10%) were excluded due to being admitted for intoxication alone rather than injury, resulting in 684 patients eligible for inclusion in the study. Males and females were equally represented. Of all injured participants, 35.7% tested positive for a psychoactive substance. An overview of the study population is shown in Table 2.

**Table 2:** Overview of study population

	Substance in blood or self-reported use of alcohol [n, (%)]			*p* value	Missing (n, %)
	Detected	None detected	Total		
Admitted due to injury	244 (35.7)	440 (64.3)	684 (100)		0
Sex					
Male	146 (36.7)	252 (63.3)	398 (100)	0.421	4 (0.6)
Female	95 (33.7)	187 (66.3)	282 (100)		
Age group					
18–34	60 (34.3)	115 (65.7)	175 (100)	0.442	16 (2.3)
35–64	99 (38.2)	160 (61.8)	259 (100)		
> 64	77 (32.9)	157 (67.1)	234 (100)		
Time of day					
Day shift (07–15)	86 (47.3)	96 (52.7)	182 (100)	< 0.001	14 (2)
Evening shift (15–23)	76 (24.8)	231 (75.2)	307 (100)		
Night shift (23–07)	78 (43.1)	103 (56.9)	181 (100)		
Weekday/weekend					
Mon 0000-fri 2359	120 (27.1)	322 (72.9)	442 (100)	< 0.001	1 (0.1)
Weekend	124 (51.5)	117 (48.5)	241 (100)		
Triage					
Red	20 (27)	54 (73)	74 (100)	0.203	89 (13)
Orange	52 (42.6)	70 (57.4)	122 (100)		
Yellow	104 (34.6)	197 (65.4)	301 (100)		
Green	37 (38.1)	60 (61.9)	97 (100)		
Blue	0	1 (100)	1 (100)		
AUDIT-C					
Score > = 5	105 (43.0)	91 (20.7)	196 (28.7)	< 0.001	0
Injury severity					
MAIS ≥ 3	22 (11.3)	53 (14.5)	75 (13.4)	0.289	123 (18)
ISS ≥ 9	23 (11.8)	54 (14.8)	77 (13.7)	0.332	123 (18)
Admitted to ICU	122 (50)	214 (48.6)	336 (49.1)	0.733	0

Table 2. Overview of the study population, with age groups, time of day and week, triage and length of stay, by detection or non-detection of any psychoactive substance. For alcohol, detection encompasses both a positive sample in blood and/or self-reported use within the 6 h prior to injury. All other parameters are solely positive results in the participant’s blood sample

### Time of admittance

Participants in whom alcohol or illicit substances were detected presented equally during day and night shifts, but less often during evening shifts. Alcohol was more often identified during weekends [40.1% of patients tested positive during the weekend vs. 14.1% during weekdays (*p* ≤ 0.001)]. Participants testing positive for psychoactive medicinal drugs presented quite equally between the three parts of the 24-h period. Participants testing positive for psychoactive medicinal or illicit substances showed no major differences in presentation between weekdays and weekends (*p* = 0.237 and *p* = 0.695).

AUDIT-C ≥ 5 was more often identified during weekends than weekdays (35.3% vs. 25.1%, *p* = 0.005). AUDIT-C ≥ 5 did not correlate with presentation shift during weekdays but was at weekends more often identified in the day shift (43.9%) versus 30.5% evening and 25.6% night (*p* = 0.015).

### Admittance triage and injury severity

No significant differences in triage or injury severity (MAIS ≥ 3 or ISS ≥ 9) were identified for any psychoactive substance group in bivariable testing, nor when corrected for sex and age. AUDIT-C ≥ 5 however, was identified on admittance in less participants with high triage (red or orange) (43 (21.9%) vs in participants with lower triage levels (131 (32.8%)) (*p* = 0.006). When correcting for sex, age and MAIS, no significant difference was identified.

### Length of hospital stay and ICU service use

**Fig. 2:**
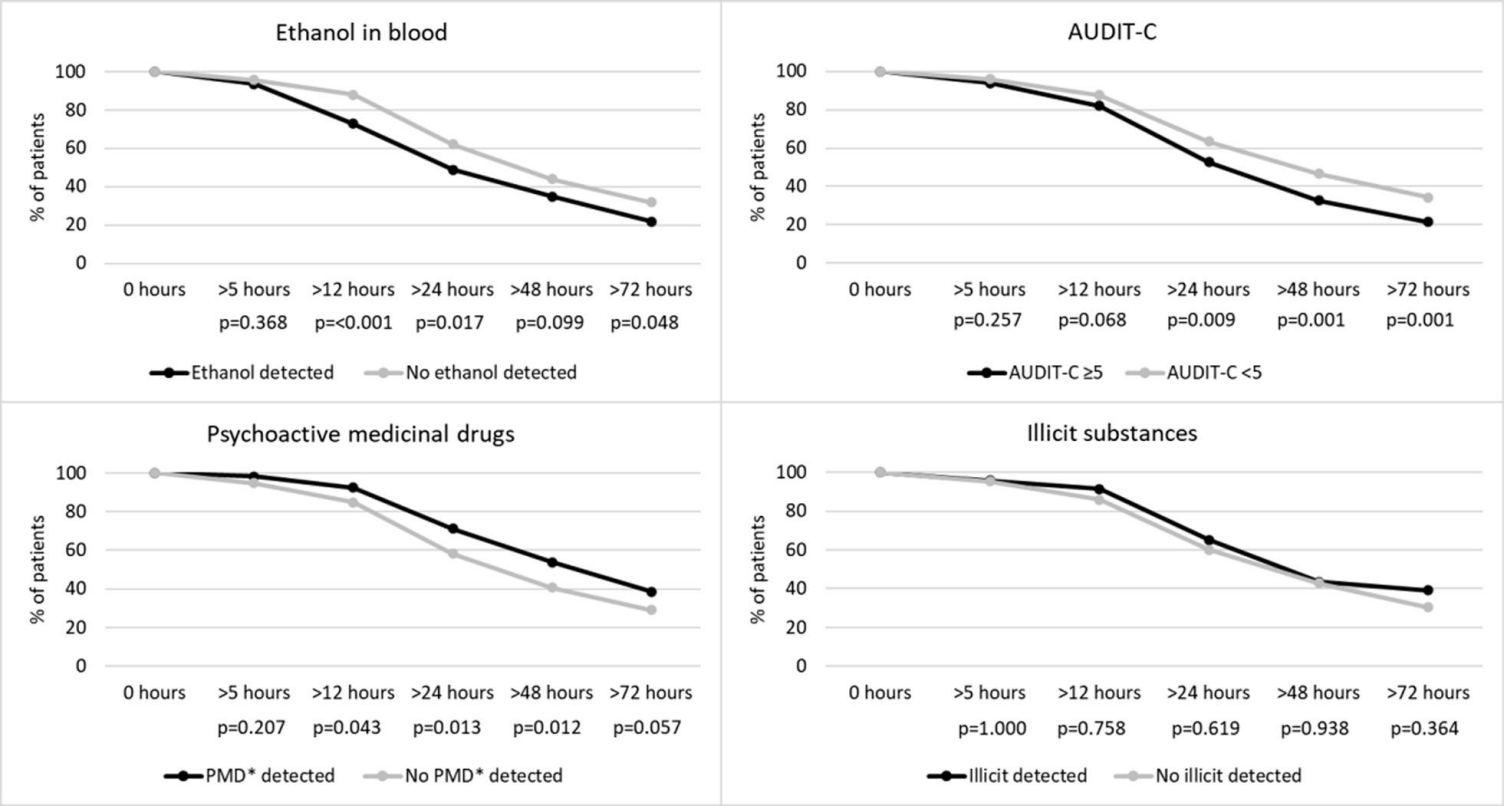
Total duration of hospital stay by identified substance and AUDIT-C. Bivariable testing of length of stay by detection or non-detection of substances and AUDIT-C. For 5 durations, length of stay is plotted for each category

The length of total hospital stay varied from 0 to > 30 days. 96 patients (14%) were admitted for ≤ 12 h. Of these 96 patients, 42 (44%) tested positive for a psychoactive substance. Participants using alcohol tended to have shorter lengths of hospital stay (Fig. 2, Table 3) while participants in whom psychoactive medicinal drugs were detected more often had longer hospital stays (Fig. 2). To account for injury severity in length of hospital stay and need for ICU services, MAIS was calculated, and when correcting for injury severity, sex and age, statistically significant association was identified for ethanol in blood and shorter stays, and illicit substances and longer stays (Table 3).

**Table 3:** Length of stay and use of ICU services

	Bivariable panel (chi^2^), n (%)					Multivariable panel^†^, n (%)			
	Detected	Not detected	Total	*p* value	Missing	Odds ratio	Confidence interval	*p* value	Missing
*Length of hospital stay*									
> 12 h									
Alcohol*	124 (79)	459 (87.9)	583 (85.9)	0.005	5 (0.7)	0.59	0.33–1.05	0.071	138 (20.2)
s-ethanol**	67 (72.8)	521 (88)	588 (86)	< 0.001	0	0.48	0.25–0.90	0.026	136 (19.9)
PMD***	96 (92.3)	492 (84.8)	588 (86)	0.043		1.72	0.58–5.07	0.328	
Illicit	21 (91.3)	567 (85.8)	588 (86)	0.758		N/A			
AUDIT- C ≥ 5	161 (82.1)	427 (87.5)	588 (86)	0.068		1.09	0.61–1.94	0.781	
> 24 h									
Alcohol*	91 (58)	319 (61.1)	410 (60.4)	0.479	5 (0.7)	1.08	0.69–1.68	0.751	138 (20.2)
s-ethanol**	45 (48.9)	367 (62)	412 (60.2)	0.017	0	0.82	0.48–1.40	0.466	136 (19.9)
PMD***	74 (71.2)	338 (58.3)	412 (60.2)	0.013		1.33	0.74–2.36	0.340	
Illicit	15 (65.2)	397 (60.1)	412 (60.2)	0.619		3.28	0.98–10.67	0.055	
AUDIT-C ≥ 5	103 (52.6)	309 (63.3)	412 (60.2)	0.009		1.12	0.73–1.72	0.617	
> 72 h									
Alcohol*	37 (23.6)	170 (32.6)	207 (30.5)	0.032	5 (0.7)	0.84	0.49–1.43	0.517	138 (20.2)
s-ethanol**	20 (21.7)	189 (31.9)	209 (30.6)	0.048	0	0.99	0.50–1.93	0.965	136 (19.9)
PMD***	40 (38.5)	169 (29.1)	209 (30.6)	0.057		1.50	0.86–2.62	0.150	
Illicit	9 (39.1)	200 (30.3)	209 (30.6)	0.364		3.31	1.09–10.08	0.035	
AUDIT-C ≥ 5	42 (21.4)	167 (34.2)	209 (30.6)	0.001		0.87	0.52–1.46	0.596	
*Admitted to intensive care unit*									
Alcohol*	77 (49)	257 (49.2)	334 (49.2)	0.967	5 (0.7)	0.91	0.60–1.36	0.633	138 (20.2)
s-ethanol**	45 (48.9)	291 (49.2)	336 (49.1)	0.965	0	0.90	0.55–1.47	0.672	136 (19.9)
PMD***	58 (55.8)	278 (47.9)	336 (49.1)	0.141		1.32	0.80–2.18	0.277	
Illicit	11 (47.8)	325 (49.2)	336 (49.1)	0.899		0.98	0.37–2.64	0.973	
AUDIT-C ≥ 5	90 (45.9)	246 (50.4)	336 (49.1)	0.288		0.99	0.66–1.47	0.952	
*ICU stay longer than 24 h*									
Alcohol*	10 (6.4)	29 (5.6)	39 (5.7)	0.701	5 (0.7)	1.11	0.48–2.58	0.802	138 (20.2)
s-ethanol**	6 (6.5)	35 (5.9)	41 (6)	0.819	0	1.59	0.61–4.12	0.339	136 (19.9)
PMD***	12 (11.5)	29 (5)	41 (6)	0.010		2.03	0.92–4.48	0.079	
Illicit	5 (21.7)	36 (5.4)	41 (6)	0.009		6.33	1.79–22.32	0.004	
AUDIT-C ≥ 5	6 (3.1)	35 (7.2)	41 (6)	0.041		0.30	0.10–0.92	0.035	

Table 3 shows associations between major substance groups, length of hospital stay and use of ICU services. Bivariable testing and multivariable testing is shown (corrected for sex, age and injury severity)

*Ethanol in blood or self-reported use of alcohol during the 6 h prior to the incident. 5 (0.7%) participants missing in bivariable testing due to missing values for self-reported alcohol use. 138 (20.2%) patients missing from multivariable analysis due to limitations in coding of injury when adhering to the guidelines from the Association for the Advancement of Automotive Medicine. Where no corresponding level of injury is described for available ICD-10 codes, no MAIS can be described. For all other variables (s-ethanol, PMD’s, illicit substances and AUDIT-C, no participants are missing from bivariable testing, but 136 (19.9%) from multivariable testing due to lack of MAIS value)

**Serum-ethanol, ethanol identified in blood sample on arrival

***Psychoactive Medicinal Drugs.

^†^corrected for sex, age and injury severity (MAIS)

N/A: Not applicable

Of all participants, 336 (49.1%) were admitted to an ICU. The OR for having an ICU stay above 24 h when testing positive for an illicit substance was high, while having an AUDIT-C ≥ 5 predicted shorter ICU stays despite similar rates of ICU admission (Table 3).

### Previous admittance history

Participants injured after using either alcohol, psychoactive medicinal drugs or illicit substances had significantly more often been previously admitted compared to substance negative participants. Participants with an AUDIT-C ≥ 5 had no difference in previous admittance history (Table 4).

**Table 4:** Previous admittance history

	Bivariable panel (chi^2^), n (%)					Multivariable panel^†^, n (%)			
	Detected	Not detected	Total	p value	Missing	Odds ratio	Confidence interval	p value	Missing
*Number of previous admittances to the health trust*									
At least one									
Alcohol*	125 (79.6)	362 (69.6)	487 (71.9)	0.015	7 (1)	2.13	1.35–3.37	0.001	21 (3.1)
s-ethanol**	71 (77.2)	420 (71.2)	491 (72)	0.234	2 (0.3)	1.64	0.95–2.83	0.075	18 (2.6)
PMD***	84 (80.8)	407 (70.4)	491 (72)	0.030		1.57	0.90–2.75	0.112	
Illicit	21 (91.3)	470 (71.3)	491 (72)	0.036		6.43	1.47–28.08	0.013	
AUDIT-C ≥ 5	137 (69.9)	354 (72.8)	491 (72)	0.439		1.47	0.98–2.20	0.065	
Three or more									
Alcohol*	82 (52.2)	234 (45)	316 (46.7)	0.112	7 (1)	1.80	1.21–2.66	0.004	21 (3.1)
s-ethanol**	45 (48.9)	275 (46.6)	320 (46.9)	0.681	2 (0.3)	1.49	0.92–2.40	0.106	18 (2.6)
PMD***	64 (61.5)	256 (44.3)	320 (46.9)	0.001		1.75	1.11–2.76	0.016	
Illicit	13 (56.5)	307 (46.6)	320 (46.9)	0.348		2.52	1.05–6.02	0.038	
AUDIT-C ≥ 5	78 (39.8)	242 (49.8)	320 (46.9)	0.018		1.11	0.76–1.63	0.590	
Five or more									
Alcohol*	62 (39.5)	169 (32.5)	231 (34.1)	0.105	7 (1)	1.81	1.21–2.72	0.004	21 (3.1)
s-ethanol**	34 (37)	201 (34.1)	235 (34.5)	0.588	2 (0.3)	1.57	0.96–2.58	0.074	18 (2.6)
PMD***	48 (46.2)	187 (32.4)	235 (34.5)	0.006		1.52	0.97–2.38	0.067	
Illicit	10 (43.5)	225 (34.1)	235 (34.5)	0.354		2.49	1.04–5.99	0.041	
AUDIT-C ≥ 5	51 (26)	184 (37.9)	235 (34.5)	0.003		0.88	0.59–1.33	0.548	

*Ethanol in blood or self-reported use of alcohol during the 6 h prior to the incident. 7 (1%) missing participants in bivariable testing. 21 (3.1%) missing from multivariable analysis. For all other variables (s-ethanol, PMD’s, illicit substances and AUDIT-C, 2 (0.3%) participants are missing from bivariable testing, and 18 (2.6%) from the multivariable panel due to lack of values

**Serum-ethanol, ethanol identified in blood sample on arrival.

***Psychoactive Medicinal Drugs.

^†^Corrected for sex and age

### Sub-group analysis: age and alcohol

Alcohol was by far the most frequently identified substance, and sub-group analysis of age was performed to identify any age-specific trends for more effective use of preventive resources (Table 5). We found that participants 18–34 years old positive for use of alcohol were often discharged after only a few hours. 35–64-year olds also had significantly higher odds of being previously admitted although this finding was only apparent when considering self-reported use of alcohol and not ethanol in blood alone.

**Table 5:** Sub-group analysis of age and selected variables

	Age group	Bivariable panel (chi^2^), n (%)					Multivariable panel^†^, n (%)			
		Detected	Not detected	Total	*p* value	Missing	Odds ratio	Confidence interval	*p* value	Missing
*Length of hospital stay more than 12 h*										
Alcohol*	18–34	29 (60.4)	94 (74)	123 (70.3)	0.079	19 (2.8)	0.404	0.18–0.91	0.028	28 (16)
	35–64	56 (83.6)	170 (89.5)	226 (87.9)	0.203		0.702	0.28–1.76	0.450	51 (19.7)
	≥ 65	36 (100)	188 (95.4)	224 (96.1)	0.191		N/A			43 (18.4)
s-ethanol**	18–34	16 (50)	107 (74.8)	123 (70.3)	0.005	16 (2.3)	0.292	0.12–0.71	0.007	28 (16)
	35–64	28 (82.4)	200 (88.9)	228 (88)	0.274		0.597	0.20–1.77	0.351	50 (19.3)
	≥ 65	22 (100)	203 (95.8)	225 (96.2)	0.324		N/A			42 (17.9)
AUDIT-C ≥ 5	18–34	53 (72.6)	70 (68.6)	123 (70.3)	0.570		1.174	0.53–2.61	0.694	28 (16)
	35–64	77 (85.6)	151 (89.3)	228 (88)	0.370		0.843	0.35–2.06	0.708	50 (19.3)
	≥ 65	29 (100)	196 (95.6)	225 (96.2)	0.250		N/A			42 (17.9)
*At least one previous admittance to the health trust*										
Alcohol*	18–34	35 (72.9)	67 (52.8)	102 (58.3)	0.016	21 (3.1)	2.482	1.19–5.16	0.015	0
	35–64	54 (80.6)	125 (65.8)	179 (69.6)	0.023		2.282	1.14–4.57	0.020	2 (0.8)
	≥ 65	31 (86.1)	166 (85.1)	197 (85.3)	0.878		1.532	0.53–4.41	0.429	3 (1.3)
s-ethanol**	18–34	23 (71.9)	79 (55.2)	102 (58.3)	0.085	18 (2.6)	2.128	0.92–4.95	0.079	0
	35–64	25 (73.5)	156 (69.3)	181 (69.9)	0.619		1.351	0.59–3.12	0.480	0
	≥ 65	19 (86.4)	179 (85.2)	198 (85.3)	0.887		1.622	0.44–6.03	0.470	2 (0.9)
AUDIT-C ≥ 5	18–34	43 (58.9)	59 (57.8)	102 (58.3)	0.888		1.110	0.59–2.08	0.744	0
	35–64	66 (73.3)	115 (68)	181 (69.9)	0.377		1.753	0.96–3.21	0.069	0
	≥ 65	26 (89.7)	172 (84.7)	198 (85.3)	0.483		2.453	0.67–8.99	0.176	2 (0.9)

*Ethanol in blood or self-reported use of alcohol during the 6 h prior to the incident

**Serum-ethanol, ethanol identified in blood sample on arrival

^†^Corrected for sex and age when analyzing number of previous admittances, supplemented with MAIS when analyzing length of stay

Injured participants above the age of 64 had significantly less positive samples with increasing age, both regarding ethanol in blood and with self-reported use of alcohol. Sex did not overall, nor in any separate age groups have any significant bearing on results. AUDIT-C ≥ 5 was significantly more often identified in men but did not in any age groups identify participants with more previous admittances or altered length of hospital stay than participants scoring < 5.

## Discussion

In this study we found that illicit substance use was associated with extended ICU stays, that participants having recently used alcohol, psychoactive medicinal substances or illicit substances all had more often been previously admitted to hospital; and that injured participants with ethanol in blood on arrival at hospital often stayed in hospital for less than 12 h. AUDIT-C ≥ 5 was associated with lower triage on admittance and shorter ICU stays.

Ethanol-positive participants less often staying > 12 h implies that ethanol in blood on arrival predicts early discharge or the participant leaving against medical advice, the latter a phenomenon already defined as a challenge in alcohol-positive trauma patients [28]. A German study from 2005 found a significant association between staying less than 24 h in the ED and admittance related to trauma and alcohol use [29], but results are conflicting as other studies have identified increased lengths of stay in the ED after use of alcohol [30].

We found no association of any substance group with needing ICU admittance. Length of ICU stay was only increased when illicit substances were identified, in line with a previous study on burn patients [31]. Overall hospital stay in users of illicit substances was mainly unchanged, as in orthopaedic patients [32], while a large trauma registry study showed that amphetamines were associated with an increased length of stay [33]. In our material, 49.1% of all injured participants in the study were admitted to the ICU, likely due to rural resource allocation, similarly noted in a study from Sweden (20.1% in rural hospitals vs. 7.8% in larger centres) [34].

Our results of all three substance groups having higher odds of previous admittance compared to substance-negative participants indicates that hospitalization, potentially regardless of reason could be an important identifier of patients with risk of future injury. Readmission has been shown to be highly predicted by the use of alcohol or drugs [28], and self-discharge is known to be a cause of readmission in trauma patients [35]. Self-discharge was not a parameter in our study, so it is difficult to define its role in our results. In one study, 29.8% of all trauma patients were admitted for any cause in the 6 months post-injury [36], indicating that prior admittance for injury could predict future re-admittance. Comorbidities and increasing age were found to influence the risk, as in Australia [37], who also cited number of prior admissions as a risk factor. Our sub-group analysis of age showed that participants aged 18–64 in whom alcohol was detected more often had been previously admitted while older participants had not. While in the > 64’s, less positive samples were identified with increasing age, alcohol status could still be used in most ages to identify patients at risk for recurrent admittance.

The decision to divide alcohol, representing the most frequent finding, into the three age groups was due to a hypothesis that e.g. young patients could be more prone to weekend binge drinking and therefore risk of injury. In a low-resource healthcare setting preventive efforts targeting all patients if only one or two easily identifiable groups are at particular risk could unduly put pressure on available preventive resources. The alcohol-positive 18–34-year olds in our study more often staying less than 12 h indicates a difference in admittance pattern despite the region’s tradition of admittance often being supported by on-call district physicians, as does the 18–64-year olds more often having been previously admitted. Particularly the first of these findings could represent unnecessary admittance while it is unknown whether mechanism of injury, self-discharge or the need for only rapid hospital treatment were the reason for a short stay.

Presentation of alcohol-related injuries during the night and weekend is as expected, but our high presentation during the daytime less so [38]. Extended transport times and different definitions of weekend could be a factor, but this still may influence how we prepare for e.g. alcohol-related injuries in a more extended period of time in rural areas.

We propose attempting identification of patients at risk of future injury, and our findings show that choice of alcohol-identifying parameter could be tailored to geography, resources and goal. Ethanol in blood on arrival predicts early discharge and therefore possible over-use of hospital services and transport, while including self-reported use of alcohol identified those with several previous admittances, which could effectively trigger follow-up if preventive systems are put in place. Future studies should focus on the connection between psychoactive substance use and more frequent hospital admittance, and why younger alcohol-positive patients ingesting alcohol have only short stays in hospital. Observance of admittances for injury in relation to psychoactive substances should be a focus at all times of day in rural hospitals.

The similarly high prevalence of psychoactive substance use in urban areas suggests some generalizability, but we don’t know whether increased levels of previous admittance are an isolated rural phenomenon. However, increased mortality in rural areas highlights important differences which so far lack in clarity. The emergency care pathway should likely to a higher degree focus on primary and secondary preventive efforts to help avoid injury in relation to alcohol, psychoactive medicinal drugs and illicit substances.

## Limitations

Patients admitted directly to the regional trauma center are not included in this material, but is in our experience a relatively infrequent occurrence. We cannot discern between prescribed and non-prescribed use of medicinal drugs or reasons for prior admittance, which begs the question whether previous admittances were due to injury, intoxication, or as we suspect, a wider range of reasons. Injury severity was coded using guidelines from the publisher of the coding system AIS, which does not cover all injury diagnoses, therefore precluding use of all participants in statistical modelling (136 unable to be coded). ICD-10 coding was not originally performed with research in mind, but all allocated codes were considered to achieve as accurate as possible a picture of each participant without violating AIS coding rules. Time of admittance can be slightly misleading due to blood sampling time being employed as a proxy for admittance, but we suspect the impact is small due to effective sampling routines in the ED. We suspect transport times to be extended due to a large part of the area’s population living hours from the two available hospitals and a high proportion of this study’s participants being admitted after more than 6 h post-injury [1], although we do not have information on method of transport or distance, which is a limitation to the study. It is difficult to define any impact of patients declining participation due to worry of detriment despite information to the contrary. While 19% of the total group of potentially eligible patients declined participation, we suggest this still offers acceptable results in the knowledge that 35.7% of consenting patients tested positive for a psychoactive substance [1].

Despite varying definitions of rurality we consider the whole of Finnmark county to be largely rural due to more similarities than differences between even the largest population centres and smaller settlements spread across a relatively large region.

## Conclusions

Triage level and injury severity were not associated with having used a psychoactive substance prior to the incident, but AUDIT C ≥ 5 was associated with lower triage on admittance and shorter ICU stays. Length of hospital stay for participants with ethanol in their blood sample was associated with more often staying less than 12 h compared to participants without ethanol in blood, a finding primarily associated with 18–34-year-olds. ICU stays were extended after illicit substance use. Use of alcohol, psychoactive medicinal drugs or illicit substances prior to the injury was significantly associated with having been admitted up to several times previously, the same being identified for self-reported use of alcohol alone, but not AUDIT-C ≥ 5. Detecting ethanol in blood on arrival did not elicit this relationship.

This study indicates that although injury severity was not impacted by psychoactive substances, higher numbers of previous admittances and differences in hospital admission times are factors preventive efforts could utilize, such as with general screening of all patients admitted to hospital using e.g. AUDIT- C to identify patients at risk of injury.

## Data Availability

The data are not publicly available due to restrictions to avoid compromise of research participant consent and privacy. The data that support the findings of this study are available on reasonable request from the corresponding author.

## Abbreviations

AIS: Abbreviated Injury Scale
ARDS: Acute Respiratory Distress Syndrome
AUDIT-C: Alcohol Use Disorder Identification Test—Consumption
CT: Computed tomography
ED: Emergency department
GCS: Glasgow Coma Scale
ICD: International Classification of Diseases
ICU: Intensive Care Unit
ISS: Injury Severity Scale
MAIS: Maximum Abbreviated Injury Scale
METTS: Medical Emergency Triage and Treatment System
PMD: Psychoactive medicinal drug
